# Biomechanical comparison of a new expandable intramedullary nail and conventional intramedullary nails for femoral osteosynthesis in dogs

**DOI:** 10.1371/journal.pone.0231823

**Published:** 2020-05-05

**Authors:** T. Plenert, G. Garlichs, I. Nolte, L. Harder, M. Hootak, S. Kramer, B.-A. Behrens, J.-P. Bach

**Affiliations:** 1 Small Animal Clinic, University of Veterinary Medicine Hannover, Foundation, Hannover, Germany; 2 Institute of Forming Technology and Machines, Leibniz University Hannover, Garbsen, Germany; University Hospital Zurich, SWITZERLAND

## Abstract

Intramedullary nailing of diaphyseal femoral fractures is a commonly used treatment method in dogs because of its biological and biomechanical advantages compared to bone plating. To achieve adequate resistance of the intramedullary nail against torsional and axial compressive forces, additional application of transcortical screws is needed. As these interlocking screws represent a frequent cause of post-operative complications, a new expandable intramedullary nail (EXPN) was developed, which was designed to provide adequate fracture stabilisation without the need for transcortical fixation. The evaluation of the biomechanical properties of the new EXPN with regard to torsional, compressive and bending stability as well as direct comparison to the biomechanical properties of conventional Steinmann (STMN)- and interlocking (ILN) nails was carried out with different biomechanical test arrangements. No significant statistical differences regarding the torsional and bending resistance between the EXPN and ILN group were seen, which indicates that rotatory as well as bending stability of the innovative EXPN is similar to the conventional ILN. Nevertheless, the percentage deviation between the attempted and successfully reached physiological compressive forces was significantly higher (p = 0.045) in the EXPN group compared to the ILN group, which indicates that the compressive stability of the innovative EXPN might be weaker compared to the ILN. In summary, the new EXPN represents an interesting alternative to conventional intramedullary nails. However, in direct comparison to conventional interlocking nails, the EXPN has shown weaknesses in the neutralization of axial compressive forces, which indicates that at least biomechanically the interlocking nail seems advantageous. Further in-vitro and in-vivo investigations are required before clinical use can be recommended.

## Introduction

Due to its advantageous biomechanical properties compared to bone plating [1–10], intramedullary nailing is considered as the gold standard for treating most diaphyseal fractures in the human femur [1, 2, 9, 11–14] as well as it being a commonly used technique in canine osteosynthesis [2, 15, 16]. These advantages include placement near the neutral axis of the bone [1–5, 8, 9] as well as the larger area moment of inertia of the intramedullary nails, providing higher resistance in bending compared to plates of similar size [2, 4, 8, 17].

Besides these mechanical benefits, intramedullary nails are also reported to be biologically advantageous [4, 9, 10, 18], e. g. providing a lower incidence of postoperative infection[18]. In order to ensure sufficient stability against torsional and axial compressive forces, the most commonly used intramedullary nails are interlocking nails (ILN), which are dependent on the insertion of transcortical screws or bolts [2, 8, 16]. Even though various studies highlighted an excellent rate of success for the use of interlocking nails in dogs [2, 5, 10, 11, 19, 20], the application of transcortical screws also represents a major cause of complications and implant failure in canine osteosynthesis [4, 8–10, 16, 20–22], such as screw deformation and breakage [5, 6, 21] as well as weakening of the bone strength due to drilling holes needed for screw application [8]. Furthermore, if screw removal is indicated, the empty holes act as stress concentrators, which severely decrease the torsional strength of the bone [8]. In addition, the screw holes significantly weaken the rod, which may lead to nail breakage [10].

These concerns regarding screw application led to the development of a new expandable intramedullary nail (EXPN) for treating diaphyseal fractures of the canine femur, which was biomechanically evaluated in the present study. The purpose of the new EXPN was to display the good biomechanical and biological properties of conventional ILN without its disadvantage of the necessity to apply transcortical screws. The EXPN consists of several expandable segments that extend after nail insertion has been accomplished, leading to intramedullary fixation of the bone-nail-interface.

The aim of this in-vitro study was to examine the biomechanical properties of the EXPN in the canine femur as well as compare them with a conventional Steinmann nail (STMN) and regular ILN. For this purpose, the implants were exposed to compressive, torsional and bending forces of physiological and supraphysiological magnitude.

## Materials and methods

### Specimen collection and preparation

Thirty-two femora from one skeletally immature dog and 31 mature dogs, which had been euthanised or had died of causes unrelated to this study, were collected, wrapped in saline-soaked towels and stored at -20 °C until testing.

No approval of an animal research ethics committee was required, because only femora from dogs euthanised due to medical reasons not related to this study were used. The cadaveric femora were collected after permission of the patients' owners at the Small Animal Clinic, University of Veterinary Medicine Hannover, Germany. Exclusion criteria for specimen selection was excessive femoral curvature.

Before carrying out nail insertion, a transverse or short oblique (approximately 30 degrees perpendicular to the longitudinal axis of the femur) osteotomy at the level of the mid diaphysis was performed with an oscillating saw.

For compressive testing of transverse-fractured specimens, fracture treatment was successfully completed while maintaining a 5 mm gap at the fracture site. In all other cases, the aim was to achieve gap closure to ensure bone-to-bone contact of both fracture segments.

### Composition and functionality of the EXPN

The EXPN was constructed of stainless-steel 316L and developed at a company experienced in developing veterinary biomedical implants (Innoplant Medizintechnik GmbH, Hannover, Germany).

**Fig 1:**
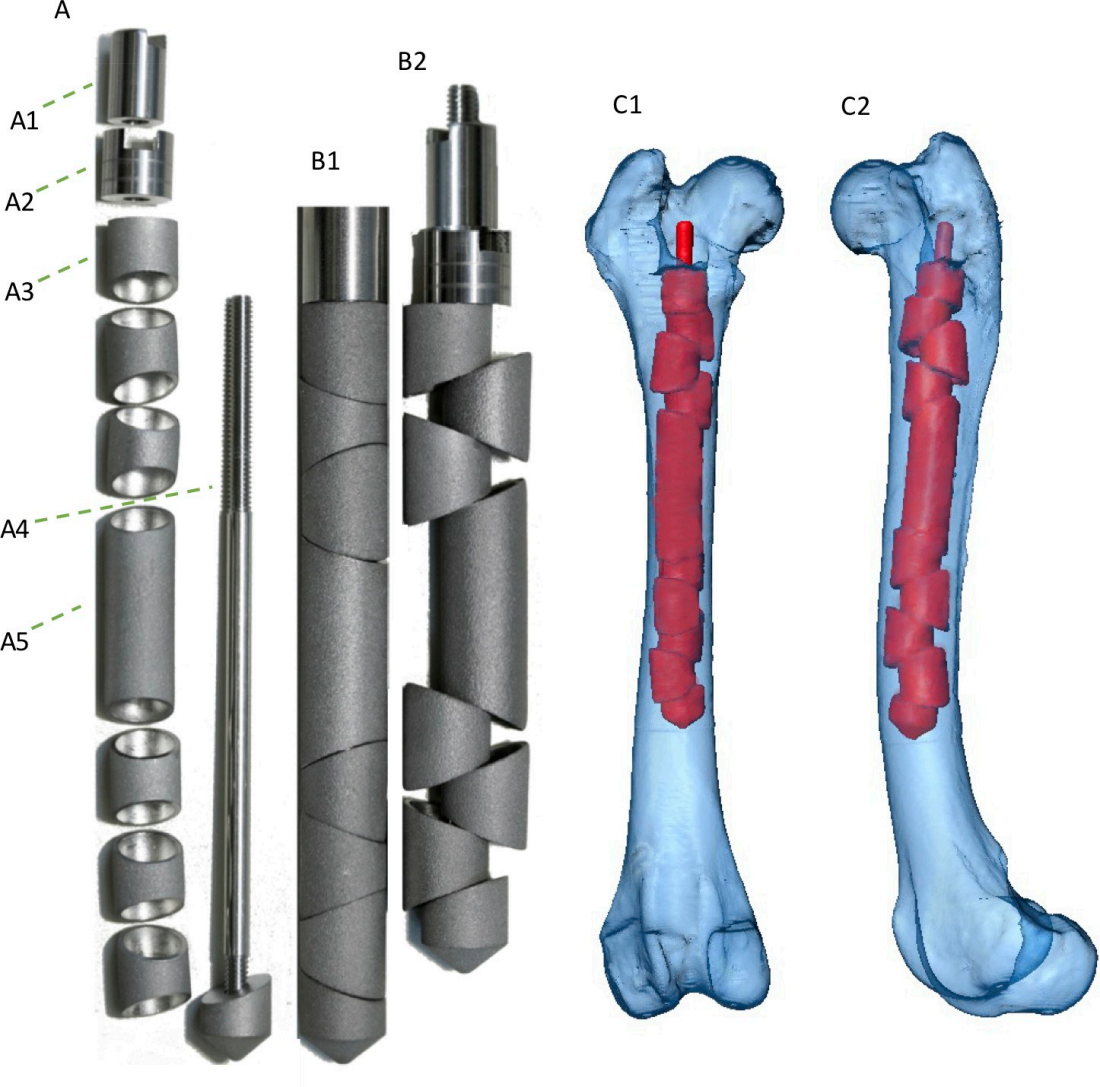
Expandable nail: Composition and 3D-model of the implanted innovative EXPN. (A) Composition of the newly expandable nail. (A1) End cap (locking nut) of the EXPN, which prevents the tension nut (A2) from loosening. (A2) Tension nut, that permits the expandable segments (A3) to extend with increasing torque. (A3) Expandable segments, with oblique borders, which sit loosely on the threaded rod (A4). (A4) Threaded rod that bears all expandable segments and the bridging element (A5). The threaded part allows the tightening of the end cap (A1) as well as of the tension nut (A2). The distal non-cutting tip is fixed with the rod. (A5) Bridging element, which links the fracture segments to ensure equal distribution of the incoming forces alongside the femoral axis.(B1) Expandable nail without the end cap and tension nut, with expandable segments fastened together on the implant’s inner casing, avoiding early expansion of the elements to allow unproblematic insertion of the nail. (B2) Expanded nail with tightened tension nut and end cap (same nail as in picture B1), demonstrating shortening of the initial nail length.

The composition of the EXPN is presented in Fig 1.

To ensure easy insertion of the EXPN into the medullary cavity, the loose expandable segments were initially lined up on an implant inner casing, providing a straight nail without any edges.

After insertion, the self-locking capabilities of the nail were initiated according to the manufactures guide, whereby the expandable units were forced against the cancellous and cortical bone to match the endosteal dimensions and fill the medullary cavity.

After expansion, the expandable segments should provide high contact pressure with the inner femoral, which might result in adequate compressive, rotatory and bending stability as well as fragment alignment without the need for interlocking screws (Fig 1).

### Implantation technique

The selection of an appropriately sized nail was ensured by measuring the best fitting diameter at the isthmus of the femur in a caudocranial and a mediolateral radiograph. Normograde nail insertion was carried out, followed by anatomical reconstruction of the fracture segments and locking of the implant (Fig 1C).

Additional information regarding the implantation- (S1 Video) and explantation (S2 Video) technique can be found in the supporting information.

To ensure a comparison of the biomechanical properties of the EXPN, fractured femora were also treated with conventional STMN (IMEX Veterinary, Inc., Longview, Texas, USA) as well as regular ILN (Dueland Interlocking Nail System, Innovative Animal Products Inc. Rochester Minnesota, USA) based on the surgical techniques described by McLaughlin et al. [16].

Further detailed information regarding the length and diameter of the used intramedullary nails can be found as supportive information (S1 Table).

### Radiographic examination (x-ray and computed tomography)

Prior to nail insertion, radiographs of all femora were taken in caudocranial and mediolateral orientation in order to ensure selection of appropriately sized implants. The presence of osteophytes at the femoral neck was rated in a semi-quantitative fashion (0—no osteopyhtes to 3—severe osteophytes). Femoral measurements were carried out using the easyIMAGE software (Vers. 8.0.0.19/R7, VetZ GmbH, Hannover, Germany). The femoral lengths were measured in caudocranial projection from the most proximal extremity of the greater trochanter to the most distal part of the lateral condyle. In addition, the diameter of femoral isthmus and cortical thickness in both mentioned planes were measured. The corticomedullary index (CMI) of all femora was calculated using the isthmus diameter and surrounding cortical thickness (medial + lateral portion) [23], measured in the caudocranial radiograph: $CMI=\frac{cortical\;thickness}{isthmus\;diameter}$ [24].

Furthermore, the canal flare index (CFI) was evaluated in a quantitative fashion. In accordance with previous studies [23, 25], the CFI was investigated as a ratio of inner medullary width at the level of the proximal extremity of the lesser trochanter to the inner medullary width at the level of the canal isthmus. In the present study, radiographs in a caudocranial projection were used for measuring the CFI. Moreover, femoral curvature was evaluated in a mediolateral radiograph in a semi-quantitative fashion (1—minor bowing to 6—strong bowing).

**Fig 2:**
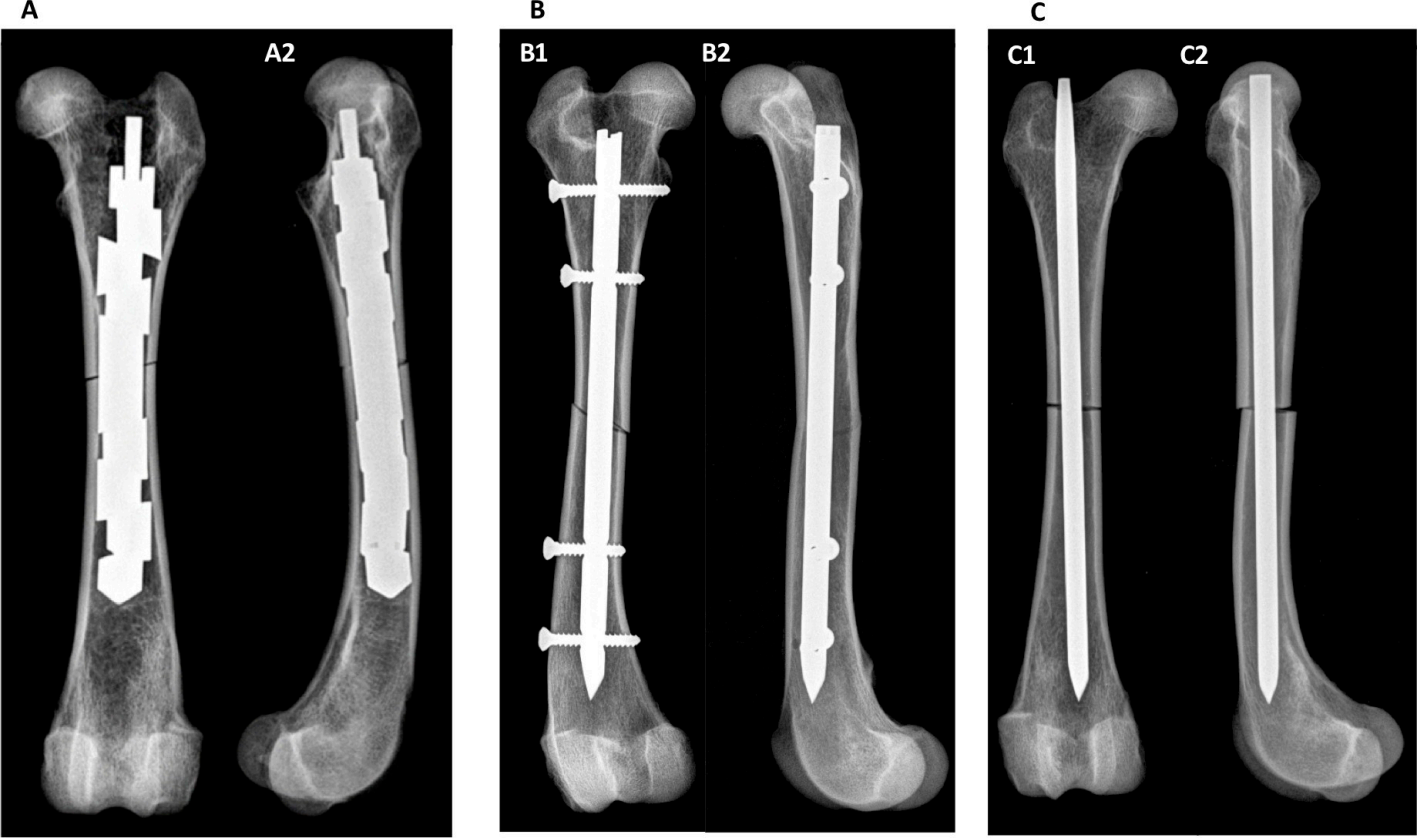
Radiographs of canine femora with implanted (A) EXPN, (B) ILN and (C) STMN. (A) Caudocranial (A1) and mediolateral (A2) radiograph of a femur with an oblique fracture and implanted and expanded EXPN. (B) Caudocranial (B1) and mediolateral (B2) radiograph of a femur with oblique fracture and implanted ILN.

In addition to this, biplanar radiographs and computed tomographic (CT) images were taken from all femora after nail insertion (Fig 2) as well as after biomechanical testing for selected specimens to verify correct nail placement and to determine whether fractures or smaller defects had been caused by nail insertion or mechanical testing.

Moreover, the maximum increase in the initial diameter of the EXPN was radiographically evaluated after successful nail implantation. Therefore, the largest diameter between two adjacent expandable segments was measured.

Additionally, the shortening of the initial nail length due to the expansion process was also measured using radiographs.

Fracture reduction was evaluated in a semi-quantitative fashion (1—nearly anatomical reduction of the fracture ends to 6—high degree of fragment displacement).

Radiographs were carried out with ‘Philips Bucky Diagnost’ (Philips Medical Systems DMC GmbH, Hamburg, Germany), at an exposure of 52 kilovoltage (peak) and 4.6 to 5.1 milliampere seconds depending on femur size. CT-images were obtained with ‘Philips Brilliance 64‘ (Philips Medical Systems DMC GmbH, Hamburg, Germany) at constant settings of 120 kV and 145 to 150 mAs depending on femur size.

### Biomechanical testing

The biomechanical properties of the bone-nail constructs were investigated using non-destructive (physiological) eccentric axial compressive (sample size n = 15) and torsional tests (sample size n = 14), as well as destructive (supraphysiological) maximum force tests for bending (sample size n = 6) and torsional loadings (sample size n = 5).

For the non-destructive testing groups, the specimens were loaded with physiological forces for axial compressive (recorded in Newton (N)) as well as torsional testing (torque, recorded in Newton metres (Nm)).

The physiological forces represent the loading conditions dogs are exposed to in normal motion and thus depend on the dogs’ bodyweight. These forces were determined in previous examinations by multi-body simulation of the physiological loads of a dog, whose kinematic and kinetic data were determined in gait analyses [26, 27]. Afterwards, the forces, which are physiologically acting on the femoral bone, were calculated by means of a multi-body simulation using the software AnyBody^™^ (AnyBody Technology A/S, Denmark). These loads represent the basis of the forces and moments to be assumed for the biomechanical tests. However, the model was based on the data of a single dog with a certain weight. As the femora of the tests originated from dogs covering different body weights, the loads had to be scaled to the considered weights. Thus, the applied test forces and moments were calculated using a scaling factor in relation to the corresponding body weight of the dog [27]. This scaling factor was determined by linear interpolation. Thus, the physiological compressive force F is scaled and calculated using Eq 1:

$$F=19,35\frac{N}{\mathrm{kg}}\times \mathrm{weight}\;(\mathrm{kilogramme}\;(\mathrm{kg}))$$ Eq 1

The physiological torsional moment is calculated in the same fashion using Eq 2:

$$T=0,05\frac{\mathrm{Nm}}{\mathrm{kg}}\times \mathrm{weight}\;(\mathrm{kg}).$$ Eq 2

Two different machines were used for biomechanical testing. The torsional and compressive loads were carried out on a tensile testing machine S100/ZD (Dyna-Mess Prüfsysteme GmbH, Aachen, Germany). A position-controlled Wolpert Testatron tensile tester (Amsler Otto Wolpert-Werke GmbH, Ludwigshafen, Germany) was used for the bending tests.

The clamping device of the S100/ZD was modified according to the type of load. The testing device was path-controlled and pressure is applied to the bone as a result of the continuous upward movement of the clamp. The resulting force was measured by a load cell. Additionally, the upward movement of the clamp (displacement) was recorded. The displacement constituted a combination of yielding of the bone-nail construct to the resulting force as well as the stiffness and mechanical play of the testing machine consisting of many different components.

The torsional testing loads were applied by using a ball screw drive, transforming the translational axial displacement into a torsional motion.

The distal end of the femur, which was cast in polyester resin, was fixed in the container with screws. The proximal part of the femur was fixed in the clamp. It has to be noted that as rotation of the container occurs due to axial shortening of the testing device, slight compressive forces are added to this type of loading configuration as well. Therefore, an application of isolated torsional forces was not possible with this set-up.

For axial compressive tests, the container was removed and a flange was used for force transmission. The axial load was applied eccentrically to the femoral head, representing the in vivo loading configurations [15]. It has to be noted that due to this eccentric load transmission, the femora were also affected by bending forces [8], depending on the degree of femoral curvature and neck-shaft angle.

In order to carry out tests under a bending load, the femur was placed horizontally on the three-point bending test device within the Wolpert Testatron testing machine. The testing force was applied perpendicular to the femoral axis at the level of the mid-diaphysis by a punch with a punch velocity of 0.05 mm/s.

In the non-destructive tests, loading of the femora was terminated when the physiological compressive force (F) or the physiological torque (T) was reached or failure occurred. In the destructive tests, however, loading was continued until failure occurred. Failure was defined as a visible secondary fracture of the bone, a clear displacement (more than 5 mm for compressive tests or more than 5° for torsional tests) of the fracture segments or continuous stagnation or decrease in the measured force illustrated by the load-displacement curve. The loading velocity was *ω* = 0.72 rad/s or v = 0.05 mm/s for torsional and compressive tests, respectively.

After testing had been completed, the degree of rotation (°) of the bone segments at the fracture site was measured quantitatively. Furthermore, any shift, opening or compression of the bone segments was evaluated in a semi-quantitative fashion (0—no to 6—very high shift/opening/compression). Shift was defined as horizontal displacement, opening (movement of both bone segments into opposite directions) and compression (movement of both bone segments towards each other) as vertical displacement.

In addition, the occurrence of secondary femoral fracture due to biomechanical testing was checked visually and by means of radiography.

### Statistical analysis

For statistical data analysis, the software SAS 9.4, using the “SAS Enterprise Guide” version 7.15 (SAS Institute Inc., Cary, North Carolina, USA) was used.

The mean values, median, range and standard deviations were calculated for each parameter.

The investigation on the normal distribution of the parameters was conducted by the Shapiro Wilks test and visual assessment of qq-plots.

In addition, contingency tables were compiled and evaluated for dichotomous parameters. Depending on sample size, the chi-square test or Fisher-Yates test was used to check for statistically significant associations between the variables.

Moreover, logistic regression analysis was performed to investigate associations between dichotomous and quantitative parameters.

Examination of morphological (isthmus diameter, CFI, CMI and femoral curvature) differences of the specimens between the three implant groups as well as displacement of the testing device, percentage deviation between the calculated and actual reached physiological forces, maximum reached torque and force (supraphysiological torsional tests and destructive bending tests) was carried out using the two-sample students-t test for normally distributed parameters or rather the Wilcoxon-2 sample test for non-parametric data.

The level of statistical significance was set at p <0.05.

## Results

### Specimens (implant-bone constructs)

In all specimens, nail insertion was performed by opening the proximal part of the medullary cavity. Additional reaming of the diaphyseal part of the femora for enlarging the medullary canal was not necessary.

No statistically significant differences regarding the endosteal femoral morphology (isthmus diameter, femoral curvature, CMI and CFI) of the used specimens between the three different implant groups were found.

#### Expandable nails

A total of 20 EXPN were implanted (diameter: median 10 mm; length: median 120 mm; S1 Table). Two femora were fractured during the expansion process of the EXPN (Nos. 8 & 10; S2 Table), but were not excluded from biomechanical testing. One of these fractures was stabilised with cerclage wire initial to biomechanical testing. In one specimen the fracture occurred as a result of the expansion process. Closure of the artificially prepared fracture was considered to be successful if no continuous fracture gap remained after implant insertion. This was achieved in all cases (15/15), where complete closure of the fracture gap was intended.

The expansion mechanism of the EXPN resulted in shortening of the nail, depending on the number of expandable units and the degree of expansion (up to 10 mm shortening; S2 Table).

#### Interlocking nails

Seven interlocking nails (diameter: median 6 mm; length: median 165 mm) were used. In all fractures treated with interlocking nails, four transcortical screws (two screws each at the proximal as well as the distal segment) were placed. One bone (specimen no. 25) showed an approximately 2 cm longitudinal fracture running from the proximal transcortical screw of the distal bony fragment to the osteotomy gap (S1 Fig). The specimen was not excluded from biomechanical testing and no cerclage wire was attached. Gap closure was attempted in four specimens and was successfully accomplished in two cases (S2 Table).

#### Steinmann nails

Five STMN (diameter: 5 mm; length: median 140 mm) were inserted. Gap closure was achieved in 4/4 cases.

Additional radiographs that were obtained after nail insertion indicated that all nails had been placed in the intended position.

### Biomechanical testing

#### Physiological torsional testing

**Fig 3:**
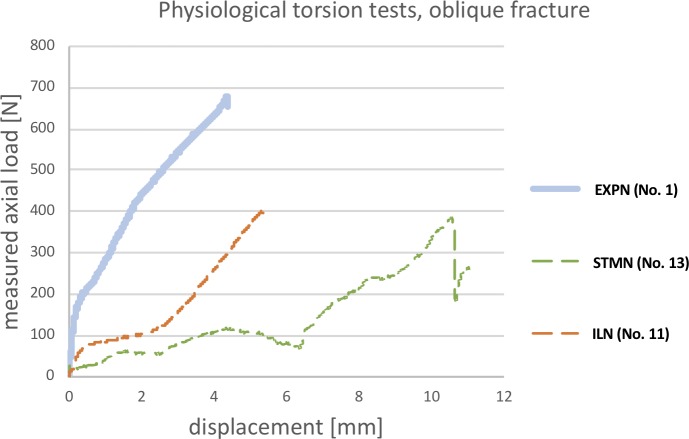
Load-displacement curves of physiological torsional testing (one example of each implant type). The line graph illustrates the amount of axial load affecting the bone-nail interface (y-axis) in relation to the shortening/displacement (x-axis) of the testing device. In the torsional test, this curve does not represent the actual acting torque.

Fig 3 illustrates the load-displacement curves of the different implants. It has to be noted that these curves present the displacement (shortening) of the testing device related to the measured force. However, this force does not represent the actual acting torque on the specimen, but rather indicates the degree of load resistance of the specific construct. The EXPN gradient compared to the remaining implants was significantly higher in the initial period of testing. Nevertheless, after a moderate amount of displacement, the ILN gradient was about the same as the EXPN. In contrast, the STMN curve did not rise until a high degree of displacement was present.

In this testing set-up, there were no significant differences between the implant groups and the displacement of the testing device.

The percentage deviation between the calculated and successfully reached physiological forces tended to be higher in the STMN group compared to the EXPN group (p = 0.055).

#### Expandable nails

In 8/10 cases, the bone-implant constructs reached the calculated physiological torque. Only in one specimen was minor rotation (approximately 3°) of the bone fragments visible. Furthermore, in 3/10 constructs, a minor (2x) to moderate (1x) shift of the fragments at the fracture site, as well as a minor (3x) to moderate (1x) opening of the fracture gap in 4/10 specimens were present. Nevertheless, there was no evidence of gap compression. Due to testing, in 2/10 cases, a 2–4 cm longitudinal fracture starting from the osteotomy gap occurred (Table 1).

**Table 1:** General information of the used specimen and results of biomechanical testing.

Implant group^a^	Type of testing	Number of used specimens	Mean body weight (kg)	Mean femur length (mm)	Mean corticomedullary index (CMI)	Mean canal flare index (CFI)	Mean nail diameter (mm)	Mean deviation of the force^b^ (%)	Mean displacement^c^ (mm)	Fractures due to testing
EXPN	Torsion	**10**	**34.5**	211.8	0.43	2.26	**9.9**	**6.2**	3.84	**2**
ILN	Torsion	**2**	**31.1**	197.5	0.38	2.43	**7**	**0**	**2.3**	**1**
STMN	Torsion	**2**	**25.5**	172	**0.49**	2.75	**5**	**43.5**	7.25	**0**
EXPN	Compression	**10**	**31.7**	203.3	**0.46**	2.42	**9.4**	**40.3**	6.25	**4**
ILN	Compression	**3**	**31.33**	203	**0.48**	2.55	**6.67**	**0**	**4.83**	**0**
STMN	Compression	**2**	**22**	**184**	**0.52**	2.42	**5**	**57**	**10.75**	**0**
EXPN	Bending	**2**	**30**	**183.66**	**0.56**	2.45	**8**	**-**	**7025**	**0**
ILN	Bending	**2**	**28.2**	**189**	**0.47**	2.33	**7**	**-**	**4.6**	**2**
STMN	Bending	**2**	**29**	**196.3**	**0.49**	2.75	**5**	**-**	**7.6**	**1**

^a^: EXPN = expandable Nail; ILN = interlocking nail; STMN = Steinmann nail

^b^: Displays the percentage deviation between the calculated physiological force and the actual resisted force.

^c^: Displacement/shortening of the testing device including the femoral bone.

One of these bones had already obtained a fissure during nail insertion, but the fracture further expanded (approximately 4 cm) as the torque acting on the femur increased. The other fracture (approximately 2 cm) was seen in a specimen without any evidence of femoral fracture due to previous nail insertion (S2 Fig). Neither of these secondary fractured constructs reached the calculated physiological torque. Nevertheless, the construct that did fracture during nail insertion and was treated with a cerclage wire (specimen no. 8) successfully reached the physiological forces as well as showing no rotation, shift of the fragments or opening of the fracture gap.

#### Interlocking nails

In 2/2 femora with inserted ILN, the calculated physiological torque was reached. Although in both tested specimens, no rotation, shift, opening or compression of the bone fragments was visible after the testing procedure, moderate torsional shifting (approximately 3–5°) was visible during testing as torsional load was applied. In addition, in one bone-implant construct, a longitudinal fracture (approximately 3 cm) starting from the osteotomy gap was present as a result of the testing procedure (S3 Fig).

#### Steinmann nails

0/2 implant-bone interfaces reached the calculated physiological torque. In both specimens, torsional failure occurred due to approximately 5° rotation as well as in 1/2 implant-bone constructs a moderate shift of the fracture ends was visible. In both femora, a minor (1x) to high (1x) degree of gap compression occurred, this resulting in bone-to-bone contact while testing. Nevertheless, no fractures were detectable after testing.

#### Physiological compressive testing

**Fig 4:**
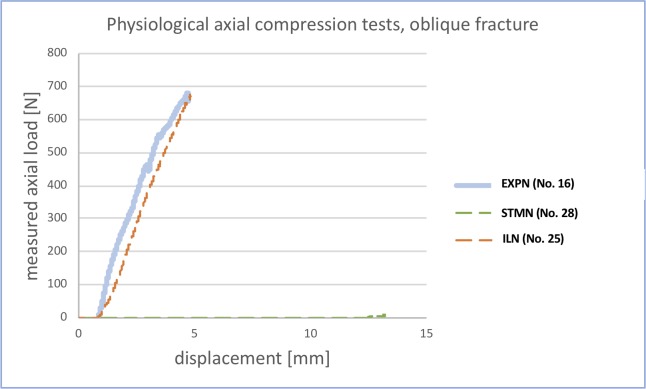
Load-displacement curves of physiological compressive testing (one example of each implant type). The line graph illustrates the amount of axial load resisted by the bone-nail interface (y-axis) in relation to the shortening/displacement (x-axis) of the testing device.

Fig 4 illustrates the load-displacement curves of selected specimens with the three different inserted nail types. The gradient of the EXPN and ILN were very similar, whereas the STMN curve did not rise at all. A higher gradient relates to a higher resistance against incoming loads.

In the compressive testing set-up, the STMN group showed a significantly higher displacement of the testing device compared to the ILN group (p = 0.049). In addition, the STMN group tended to show a higher displacement compared to the EXPN group (p = 0.05).

The percentage deviation between the calculated and successfully reached physiological forces was significantly higher in the EXPN group compared to the ILN group (p = 0.045).

#### Expandable nails

In 2/10 cases, the bone-implant constructs successfully reached the calculated physiological forces. In all tested specimens, there was no rotation of the bone fragments due to testing. Nevertheless, after testing, in 2/10 constructs, a minor shift of the fragments at the fracture site, and in the same specimen, a minor opening of the fracture gap were present. In one implant-bone construct, a high degree of gap compression was visible, resulting in bone-to-bone contact of this specimen while testing. Furthermore, in 6/10 cases, some elastic axial deformation of the bone-nail-interface occurred (S4 Fig), this however shifting back into its original conformation with decreasing pressure. After testing, in 4/10 cases, a 2–3 cm longitudinal fracture starting from the osteotomy gap was visible. It should be noted that 3/4 of these secondary fractured specimens were present in the group with a maintained gap and showed axial deformation, whereas in only 1/4 constructs with a closed gap did fracture occur due to testing. Nevertheless, the femur of an immature dog (specimen no. 18; S2 Table) successfully reached the physiological forces as well as showing no axial deformation, shift of the fragments, opening or compression of the fracture gap.

#### Interlocking nails

In 3/3 femora with an inserted ILN, the calculated physiological forces were reached. In all tested specimens, no rotation, shift, opening or compression of the bone fragments due to testing was detectable. Furthermore, no elastic deformation or fracture of the implant-bone constructs occurred.

#### Steinmann nails

0/2 implant-bone interfaces reached the calculated physiological forces. In 1/2 specimens, a moderate degree of rotation (approximately 30°) as well as a minor shift of the fracture ends was noticeable. In both femora, there was a high degree of gap compression, resulting in bone-to-bone contact while testing. Nevertheless, no elastic deformation or fracture due to testing was present in either of the cases.

### Destructive bending tests

**Fig 5:**
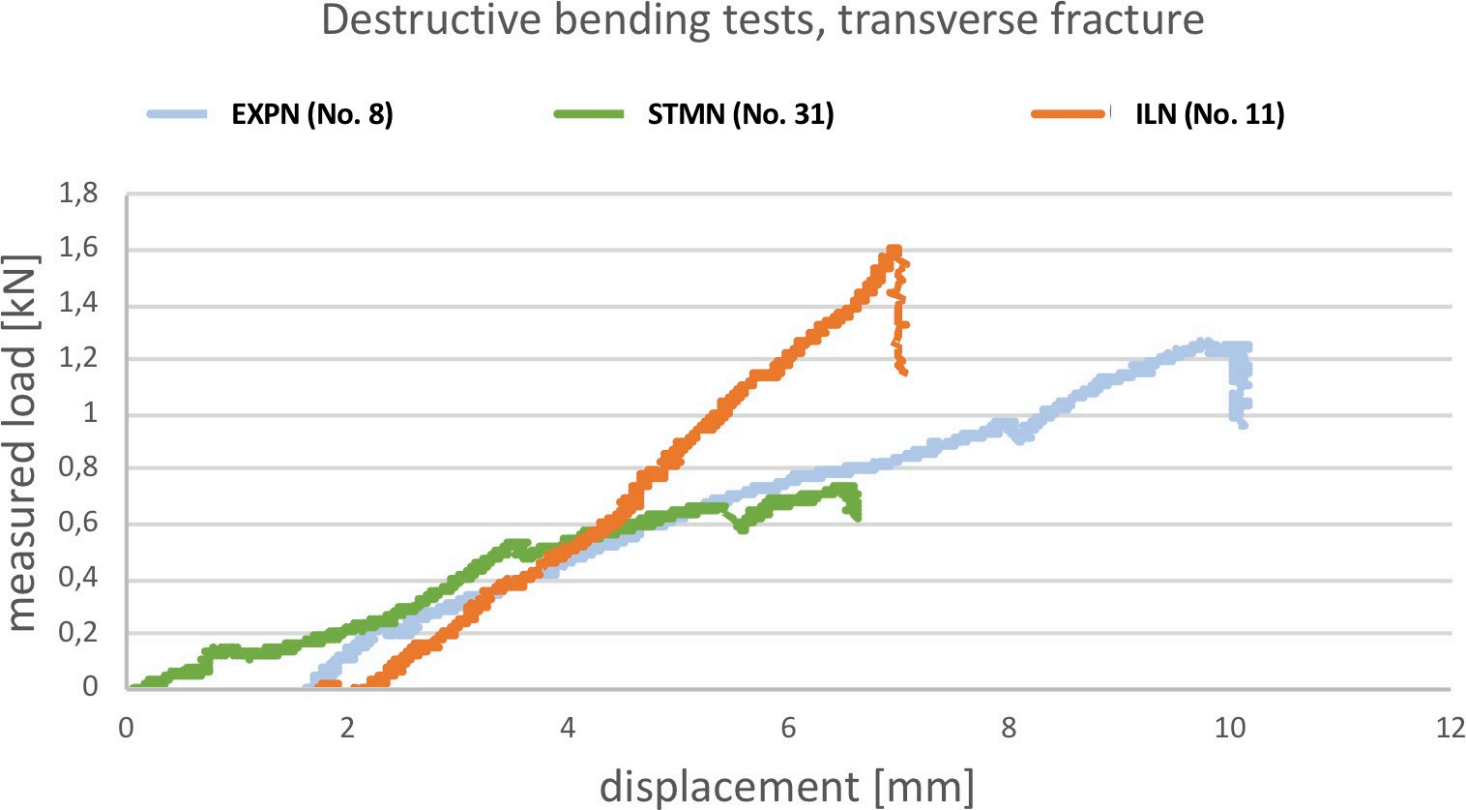
Load-displacement curves of destructive bending tests (one example of each implant type). This line graph illustrates the amount of bending load (y-axis) acting on the fracture site perpendicular to the anatomical axis in relation to the shortening/displacement (x-axis) of the punch of the testing device.

In this series, for evaluating the bending properties of the implants, specimens No. 5 & 8 were used for the EXPN, Nos. 30 & 32 were used for the ILN and Nos. 29 & 31 were used for the STMN group (S2 Table). The bending resistance of all three nail designs was similar. Increasing bending load resulted in higher bowing of all constructs, although it has to be noted that the ILN-bone-interface showed the least bending. In all tested specimens, only elastic deformation occurred, this returning to the initial conformation with decreasing bending load. Fig 5 illustrates the load-displacement curves of selected specimens of all three implants. The gradient of the ILN is slightly higher compared to the EXPN and STMN. Otherwise, the gradients were very similar to each other.

Nevertheless, both specimens with implanted ILN (S5 Fig) and 1/2 femora with inserted STMN fractured due to the applied bending load, whereas the cortical bone of both EXPN-bone constructs stayed intact.

No statistical differences between the investigated implant groups regarding the displacement of the testing device were found.

In addition, the ILN group resisted a significantly higher maximum bending load compared to the STMN (p = 0.006).

#### Supraphysiological torsional tests

**Fig 6:**
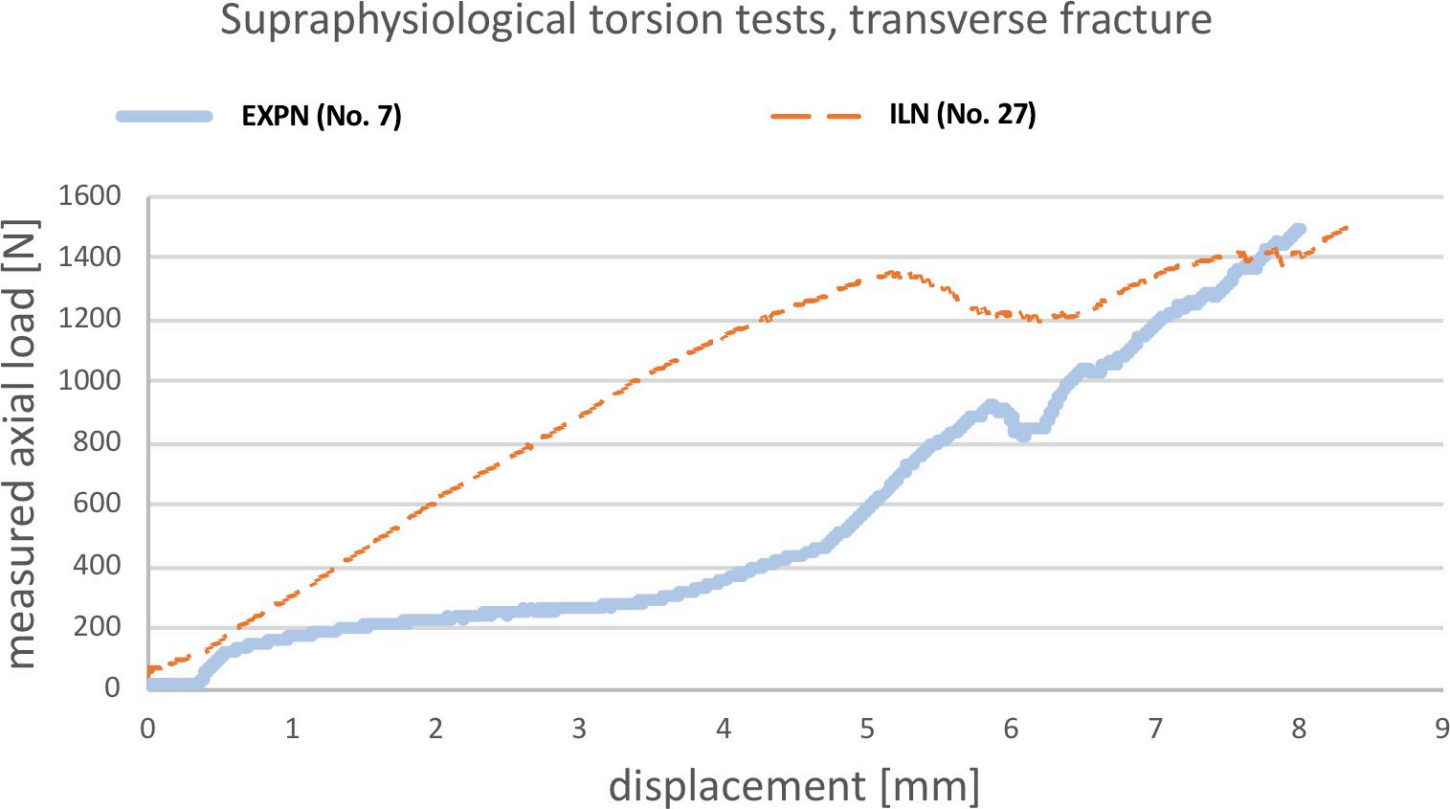
Load-displacement curves of supraphysiological torsional testing (one example of EXPN and ILN). This line graph illustrates the amount of axial load affecting the bone-nail interface (y-axis) in relation to the shortening/displacement (x-axis) of the testing device. In the torsional test, this curve does not represent the actual acting torque.

For comparison of maximum torsional resistance, three femora with inserted EXPN (Nos. 2, 4, 7) and two specimens with inserted ILN (Nos. 11 & 27) of various size, that had already successfully performed in the non-destructive tests, were used (S2 Table). As STMN had already failed to reach the physiological torque, they were excluded from this series of tests. Fig 6 illustrates selected load-displacement curves of both aforementioned nails. The initial gradient of the ILN-bone construct is higher compared to the EXPN, which initially showed a plateau without increasing load. After some increase in displacement, the gradient was about the same or even slightly higher for the EXPN.

The mean maximum torque reached in all tested specimens was 266% higher (n = 3; range: 213–333%) for the EXPN constructs (mean 4.17 Nm) and 205% higher (n = 2; range: 150–259%) for the specimens with inserted ILN (mean 3.33 Nm) compared to the specific calculated physiological torque, respectively. Nevertheless, the higher maximum torque, which was reached by the EXPN group, did not show any statistically significant differences. For the EXPN constructs, testing was terminated because of secondary fracture (1x), shift (1x) and rotation (1x) of the bone fragments. Moreover, for the ILN group, testing was terminated due to rotation (2x) of the fragments. Contrary to the EXPN, the rotation of ILN-bone constructs only affected the elastic region of the implant. Thus, they returned to their original shape with no permanent deformation when torque was released.

A statistically significant correlation was found between the occurrence of femoral fracture due to testing and achievement of the calculated physiological forces (p = 0.01).

**Fig 7:**
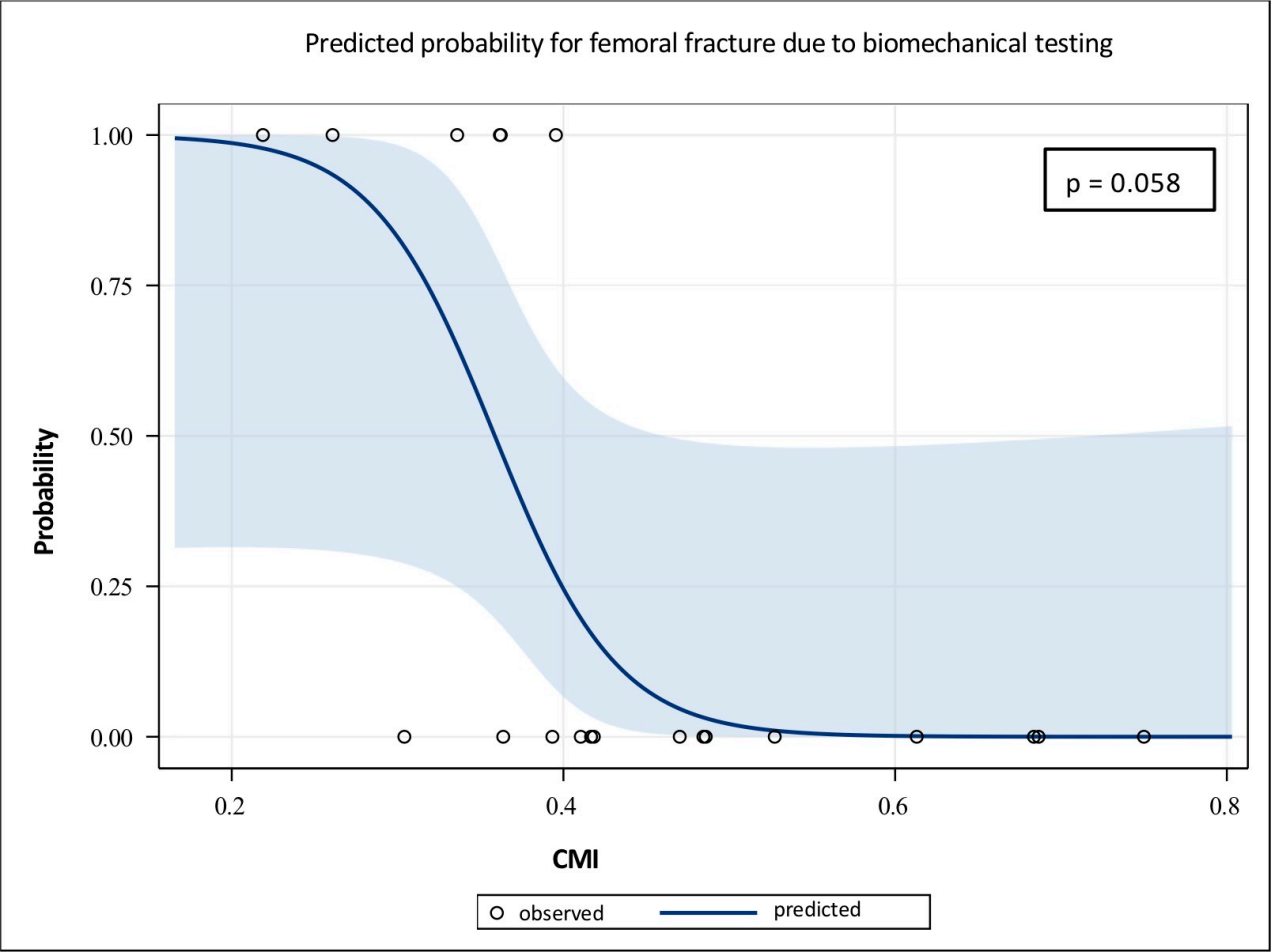
Logistic regression curve of the correlation between CMI and secondary fracture of the EXPN-bone constructs due to testing. This graph illustrates a trend in correlation between the presence of secondary femoral fracture due to testing and the corticomedullary index (CMI).

Even though no significant correlations were found between the anatomical characteristics (CFI, femoral curvature) and the outcome of biomechanical testing, there was a trend in correlation between the corticomedullary index and the risk of fracture due to testing (p = 0.058; Fig 7).

## Discussion

During normal motion of dogs, the femur is exposed to different loading types, such as compression, bending, shear and torsion [8]. Therefore, the treatment method used for stabilising femoral fractures should be able to compensate these physiologically applied forces.

In order to simulate these forces, evaluation of the biomechanical properties regarding the torsional stability of the new EXPN was carried out using a testing device, which was capable of applying rotatory forces onto the bone-nail construct.

The EXPN proved to have good biomechanical properties in neutralising torsional load. In most cases, the physiological torque was reached by the EXPN without any evidence of segment rotation or secondary femoral fracture, indicating that rigid torsional stabilisation of the fracture segments is achievable using the EXPN. In contrast, although the ILN constructs did not show any shift or rotation of the fracture segments after testing, moderate torsional shifting (approximately 4°) was visible during testing as torsional load was applied. This rotatory micromotion of the ILN, also known as torsional slack, which represents a common issue in standard ILN [4, 20], illustrated that rigid torsional stabilisation of the fracture proved impossible with this implant. Even though micromotion may improve secondary bone healing in comminuted fractures, it may lead to non-union in simple fractures (transverse/oblique) [4, 8, 28]. Nevertheless, after releasing load, the ILN-bone construct returned to its initial position.

As no significant differences regarding the percentage deviation of the calculated and reached physiological forces between the EXPN and ILN groups were seen, this might indicate that the torsional stability of the new EXPN is at least comparable to the ILN. Deviation illustrates the difference of the calculated physiological forces, which the construct should reach, and the actually reached load.

Supraphysiological torsional tests showed that the maximum torque which the nail-bone constructs withstood prior to failure was more than twice as high as the calculated physiological torque in both examined implants and was even superior (non-significantly) in the EXPN group. These results highlighted that the EXPN might even withstand torsional loads that are considerably higher than the load that is acting on the femur during normal activity of the patient.

Secondary femoral fractures occurred during testing in a few EXPN-bone constructs, in which an extremely low corticomedullary index was present. In addition, these specimens represented those constructs that could not reach the physiological torque. Moreover, even though the ILN showed good results in withstanding physiological torsional forces, despite the appearance of torsional slack, secondary fractures due to testing also occurred in this implant type. This illustrated that secondary femoral fractures due to testing might occur in conventional types of fracture treatment as well as in fractures treated with the EXPN.

Adequate torsional stability of the implant-bone construct is especially important in dogs due to their inability to restrict weight-bearing in the initial period after osteosynthesis [9]. The results of this study indicate that the capability of the new EXPN to withstand physiological torsional loads is sufficient and might even be superior to a conventional ILN due to its absence of torsional slack.

During normal motion of the dog, the femur is also exposed to bending loads [8]. In order to evaluate the bending properties of the EXPN, three-point-bending tests were carried out.

The load-displacement curves regarding the bending resistance of the EXPN were similar to both conventional implants. In addition, no significant differences were found regarding the resisted maximum bending load between the EXPN and ILN, which indicated that the EXPN provided comparable properties to withstand isolated bending forces.

Although the ILN constructs in the present study were capable of resisting a significantly higher amount of bending load compared to the STMN group, all specimens with inserted ILN fractured at a certain degree of femoral bowing. Thus, the risk of secondary fractures due to applied bending forces might be higher in ILN in comparison to EXPN and STMN.

Physiologically, the femur of a dog is also exposed to compressive forces, which are eccentrically applied to the femoral head during normal motion [8]. In order to simulate this in-vivo loading configuration, compressive loads were applied by the testing device to the femoral head as well.

The compressive testing of the EXPN showed that most constructs did not successfully reach the physiological forces. In addition, the percentage deviation was significantly higher in the EXPN group compared to the ILN, which might indicate that ILN provided better biomechanical properties compared to the new EXPN regarding compressive stability. Nevertheless, these results must be treated with caution because in all of these unsuccessful attempts, either secondary femoral fracture due to testing or axial elastic deformation of the specimen occurred, which might be a reason for the poor results in the compressive tests. Axial deformation most likely occurred because the distal end of the femur was rigidly fixed in a clamping device while axial compressive load was applied eccentrically to the femoral head. Despite these issues, compression of the maintained gap was seen in only one specimen in the EXPN group. On the contrary, in both STMN, compression of the gap occurred, which indicated that this conventional implant was incapable of providing adequate compressive stability. Supporting this thesis, displacement of the testing device, which serves as an indicator for instability of the fracture treatment, was significantly higher in the STMN groups compared to the ILN group Therefore, as the maintained fracture gaps of most EXPN constructs did not close due to the applied compressive forces, resistance to isolated axial compressive load might be better than the results may suggest even though the physiological force was not reached in most EXPN.

Another reason for the poor results of the EXPN might be the maintained gap of the transverse fractured specimen for compressive loading tests, which resulted in an unstable fracture model that represented the worst case scenario in clinical practice [4]. This was intentional to visualise gap compression, which could be seen if the implant-bone construct was incapable of providing sufficient compressive stability. The expansion process of the EXPN conducts interfragmentary gap compression, which tends to achieve gap closure and may result in better biomechanical properties.

Regarding the relatively high amount of secondary femoral fractures due to testing, it should be noted that in this study only initially frozen femora were used, which still provide adequate strength and rigidity, but lack resilience and flexibility compared to vital tissue [29]. The expandable segments might have led to high pressure against the intramedullary cortices, especially as additional external load was applied. This might have resulted in secondary femoral fractures, particularly as the resilience of the specimen was decreased by the storage process. Furthermore, all secondary fractured specimens in this group had a low corticomedullary index, which might also be a reason for the frequently seen fractures in this group.

In contrast to this, neither the STMN nor the ILN showed any axial deformation or secondary fracture due to testing. This leads to the assumption that the EXPN has limited biomechanical properties against eccentric axial compressive forces, especially if gap closure could not be achieved.

Due to the superiority of CT-images regarding accuracy compared to standard radiographs [30, 31], reliable evaluation of bone condition as well as detection of minor fractures was possible in the present study. An additional CT-image of a secondary femoral fracture, demonstrating the superiority of this modality compared to conventional radiographs can be found in the supporting information (S6 Fig).

The new expandable nail evaluated in this study was designed to combine the advantages of intramedullary nails with adequate rotational and compressive resistance without the need for transcortical fixation. The results of the biomechanical testing confirmed this ability of the new EXPN regarding its good biomechanical properties to withstand physiological torsional and bending loads. Nevertheless, the compressive stability of the EXPN showed some weaknesses.

In conventional intramedullary nailing, femoral curvature impedes the usage of this treatment method, as the rigid implant cannot adapt to moderate femoral bending, which might lead to a mismatch between the nail and endosteal shape [14]. If this mismatch cannot be compensated by further intramedullary reaming, it may lead to serious complications [13, 14].

The EXPN was capable of matching moderate femoral curvature, as the alignment of the expandable segments was, to some degree, able to adapt to the space within the medullary cavity.

In addition, as transcortical screw application was not needed, stabilisation of the fracture was quickly achieved. In a clinical practice, this advantage should reduce the duration time of surgical treatment compared to the usage of conventional ILN, as nail insertion is possible without further exposure of the femoral diaphysis. This property of the new EXPN may be advantageous compared to interlocking nailing, as the less invasive surgical approach may result in a decrease in soft-tissue damage, thus causing less disruption of the extraosseous blood supply [16, 32]. Additionally, this may contribute to early fracture healing [20, 32, 33].

The results of this study showed that the risk of femoral fracture due to biomechanical testing tended to correlate with the corticomedullary index, although no statistical significance was reached. This leads to the conclusion that the new EXPN should be used with caution if only a small amount of cortical bone is present. Therefore, careful consideration must be given when using the EXPN in canine breeds which tend to have relatively little cortical bone volume like German Shepherd dogs (S1 Table). In the present study, only one femur of this breed was included and as expected this specimen showed a low corticomedullary index, resulting in secondary femoral fracture during testing.

A disadvantage in the implantability of the EXPN was that no concrete information regarding the acting inner cortical forces applied by the expandable segments was available during the expansion process. This might be an issue especially in femora with low corticomedullary indices.

Implantation of the innovative EXPN can be rated as relatively safe regarding the few incidences of secondary femoral fractures due to nail insertion or the expansion process.

Usage of the EXPN provides shortening of the nail length, which might result in gap closure and interfragmentary compression (bone-to-bone contact) similar to the function principle of a dynamic compression plate. This may contribute to load sharing and increased friction between the fragments, increasing the mechanical stability of the construct as well as minimising micromotion, which may lead to primary osteonal fracture repair [8, 34]. The results of this study highlighted that bone-to-bone contact was successfully accomplished in all intended specimens.

### Limitations

This study was based on an in-vitro approach, aiming at evaluating the biomechanical properties provided by a new expandable nail. Nevertheless, there are several parameters that cannot be fully investigated on an in-vitro basis and that therefore require further in-vivo investigations to assist with the final evaluation as to whether the new EXPN is suitable for clinical use. These parameters include: the duration until union of the fracture segments occurs or potential complications such as non-union, which might eventually occur if torsional and axial micromotion at the fracture line cannot be sufficiently avoided; the appearance of necrosis or loss of bone density due to intramedullary cortical pressure caused by the expandable segments, which may lead to periprosthetic fractures; the decrease in blood supply due to the expansion process of the nail; the duration until the hindlimb is fully capable of weight-bearing; the degree of lameness in the initial postoperative period as well as potential challenges in removal of the EXPN after fracture healing; potential fretting of the SS316L material due to possible motion between the metal parts, which may lead to local inflammatory reactions.

Even though the removability of the EXPN was successfully tested during the development of the EXPN, possible difficulties may occur due to bony ingrowth into the spaces of the expandable units. Despite this potential disadvantage, bone ingrowth may also lead to secondary nail fixation, which might increase the biomechanical properties of the bone-nail interface in withstanding incoming load.

Furthermore, it has to be considered that although the eccentric compressive testing set-up represented the in-vivo loading configurations of a dog, the combination of compressive and bending loads also impeded the assessment of isolated compressive stability of the bone-nail constructs.

Moreover, for statistical comparison of the EXPN and conventionally used implants regarding the outcome of the biomechanical testing, only a small number of specimens with inserted ILN and STMN were available for testing. This might have led to insufficient statistical reliability.

Finally, the storage process of the specimens may have resulted in decreased resilience and flexibility compared to vital tissue, which might be a reason for the frequent appearance of secondary femoral fractures in the EXPN group.

## Conclusions

The results of the present study indicate that use of the EXPN is reasonably safe. Torsional and bending stability were similar to a regular ILN and can be considered to be sufficient for exposure to normal physiological strain. Although the EXPN might have illustrated limited properties regarding its compressive stability, this has to be further examined due to limitations regarding the applied testing procedure as well as storage process of the specimens.

Biomechanically, the ILN is superior to the EXPN, but the EXPN could provide biological advantages over the ILN, such as the preservation of soft tissue, which could possibly lead to faster fracture healing and a lower infection rate.

Since not all parameters necessary for successful application of the new nail in living animals can be evaluated in in-vitro procedures, further testing of the EXPN is necessary before its use can be considered in veterinary practice.

## Supporting information

S1 Video Nail insertion and expansion process of the EXPN. Nail insertion is carried out according to the implantation technique described in the manuscript.
(MP4)

S2 Video Explantation technique of the EXPN. The explantation of the nail is carried out using the implant’s inner casing. In the first step, the locking nut (not presented in the video) and the tension nut are loosened and removed. In the next step, the implant’s inner casing is applied to the threaded rod of the EXPN, picking up the expandable units, aligning them in a straight line to ensure easy nail removal. In the final step, the fully inserted inner casing is fixed and then the EXPN is removed.
(MP4)

S1 Fig CT-image of an interlocking nail-bone construct after nail insertion. This CT-image represents a femur (specimen no. 25) with a secondary longitudinal fracture (red arrow), running from the proximal transcortical screw of the distal bony fragment to the osteotomy gap. This fracture occurred due to nail insertion of the ILN.
(TIFF)

S2 Fig CT-images of an EXPN-bone construct after nail insertion (A) and after torsional testing (B). (A) This CT-image represents the femur (specimen no. 3) without any secondary fractures despite the artificial one. (B) This CT-image illustrates a similar image alignment of the same femur as (A) and shows a secondary longitudinal fracture (red arrow), which occurred due to torsional testing.
(TIFF)

S3 Fig Photograph of an interlocking nail-bone construct after nail insertion (A) and after torsional testing (B). (A) After nail insertion, no secondary femoral fracture is present on the cranial part of the bone (specimen no. 12). (B) After torsional testing, a secondary longitudinal fracture (black arrow) is visible due to the testing procedure.
(TIFF)

S4 Fig Photographs demonstrating the elastic deformation of an EXPN-construct during compressive testing. (A) This photograph shows the initial conformation of the femoral shape (specimen no. 18) as no compressive load was applied (starting point of the testing procedure). (B) This photograph shows a minor elastic deformation of the femur. It was taken when the physiological force has been successfully reached (time of the highest compressive load acting on the femur). Afterwards, the bone-nail construct shifted back into its original conformation with decreasing pressure (not shown in the photograph).
(TIFF)

S5 Fig CT-image of an interlocking nail-bone construct after the bending test. This CT-image illustrates two longitudinal fractures (red arrows) of a femur (specimen no. 30) that occurred due to bending testing.
(TIFF)

S6 Fig CT-image (A), biplanar radiographs (B) and a photograph (C) of an EXPN-bone construct after nail insertion. This figure illustrates the superiority of CT-images in detecting small longitudinal fractures compared to conventional radiographs. Therefore, in the CT-image (A) and photograph (C) of the femur, a longitudinal secondary femoral fracture is visible, whereas in both radiographs (B), no evidence of this fracture is given. (A) This CT-image represents the femur (specimen no. 8) with a secondary longitudinal fracture (red arrow) due to the expansion process of the nail, treated with a cerclage wire. (B) Caudocranial and mediolateral radiographs of the same secondary fractured femur, but without any radiographic evidence of the mentioned fracture. (C) A cranial photograph of the same femur, illustrating the mentioned secondary longitudinal femoral fracture (black arrow).
(TIFF)

S1 Table Detailed information regarding signalment, anatomical variations in the used specimen and characterisation of the inserted implants. (DOCX)

S2 Table Detailed presentation of the results of physiological biomechanical testing. (DOCX)
