# Supplementary figures and images for: Biomechanical comparison of a new expandable intramedullary nail and conventional intramedullary nails for femoral osteosynthesis in dogs
Source: PLoS One. 2020 May 5;15(5):e0231823. doi: 10.1371/journal.pone.0231823 (PMC7200017; doi:10.1371/journal.pone.0231823)

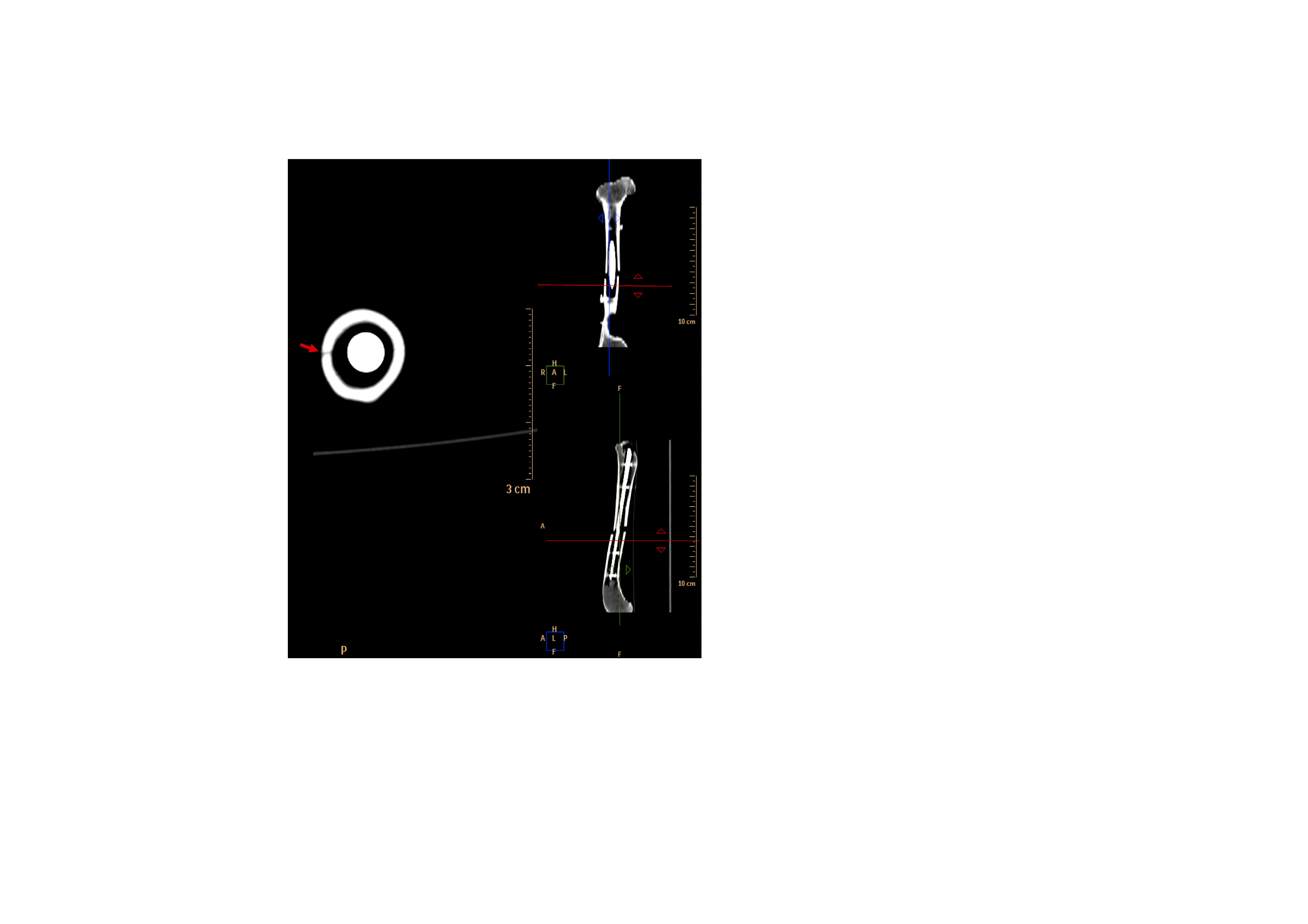

Supplement: S1 Fig — This CT-image represents a femur (specimen no. 25) with a secondary longitudinal fracture (red arrow), running from the proximal transcortical screw of the distal bony fragment to the osteotomy gap. This fracture occurred due to nail insertion of the ILN. (TIFF) [file pone.0231823.s003.tiff]

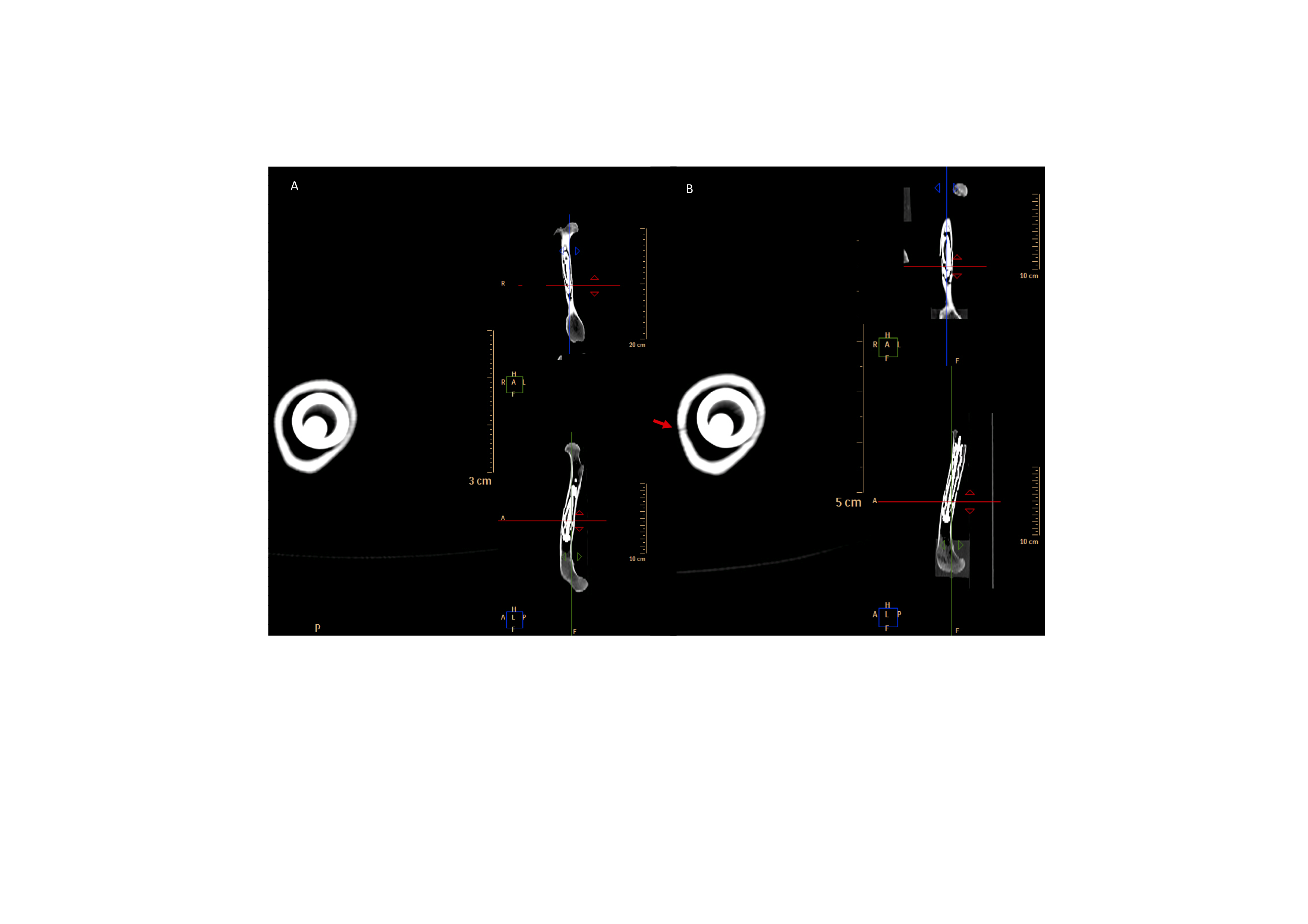

Supplement: S2 Fig — CT-images of an EXPN-bone construct after nail insertion (A) and after torsional testing (B). (A) This CT-image represents the femur (specimen no. 3) without any secondary fractures despite the artificial one. (B) This CT-image illustrates a similar image alignment of the same femur as (A) and shows a secondary longitudinal fracture (red arrow), which occurred due to torsional testing. (TIFF) [file pone.0231823.s004.tiff]

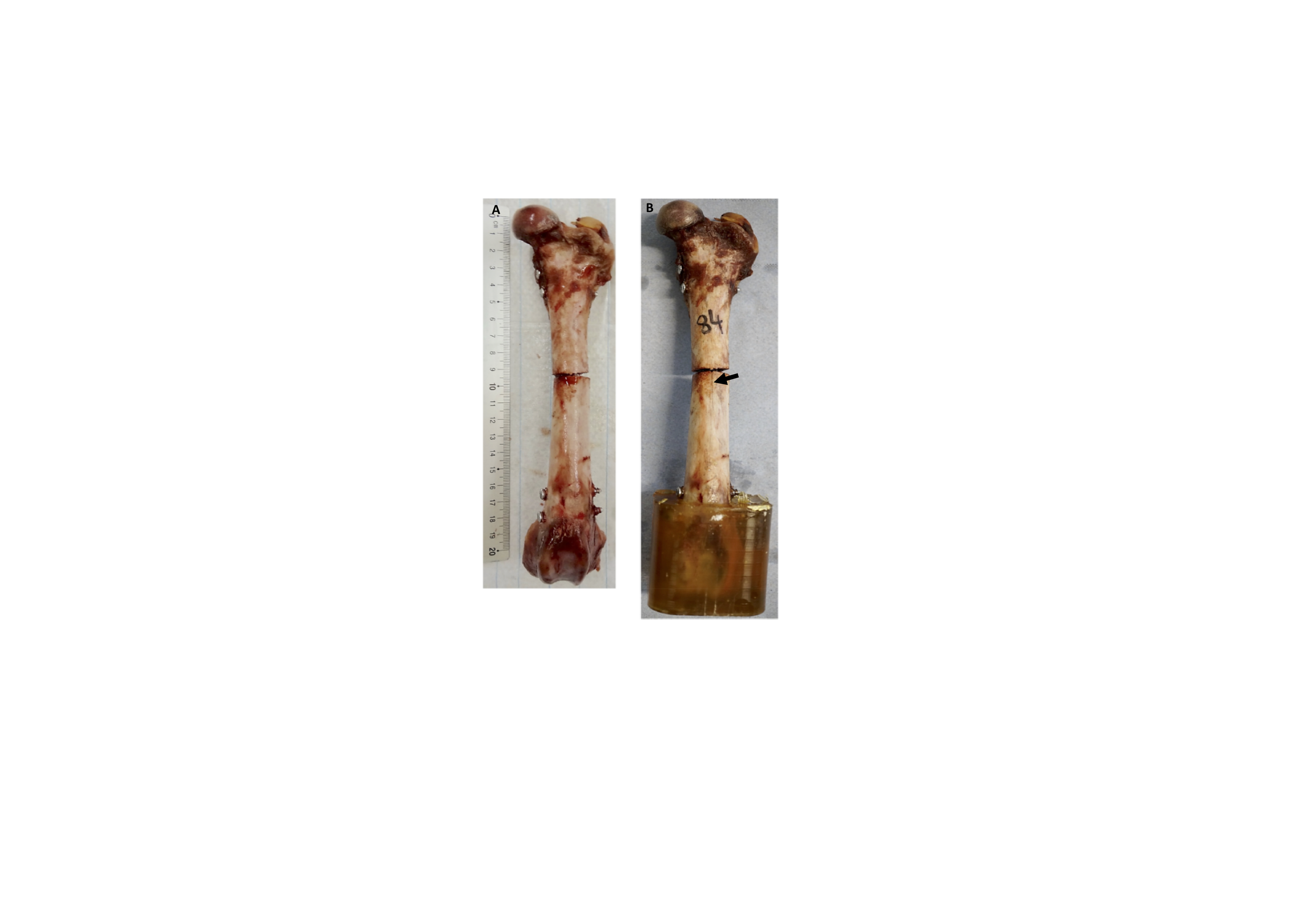

Supplement: S3 Fig — Photograph of an interlocking nail-bone construct after nail insertion (A) and after torsional testing (B). (A) After nail insertion, no secondary femoral fracture is present on the cranial part of the bone (specimen no. 12). (B) After torsional testing, a secondary longitudinal fracture (black arrow) is visible due to the testing procedure. (TIFF) [file pone.0231823.s005.tiff]

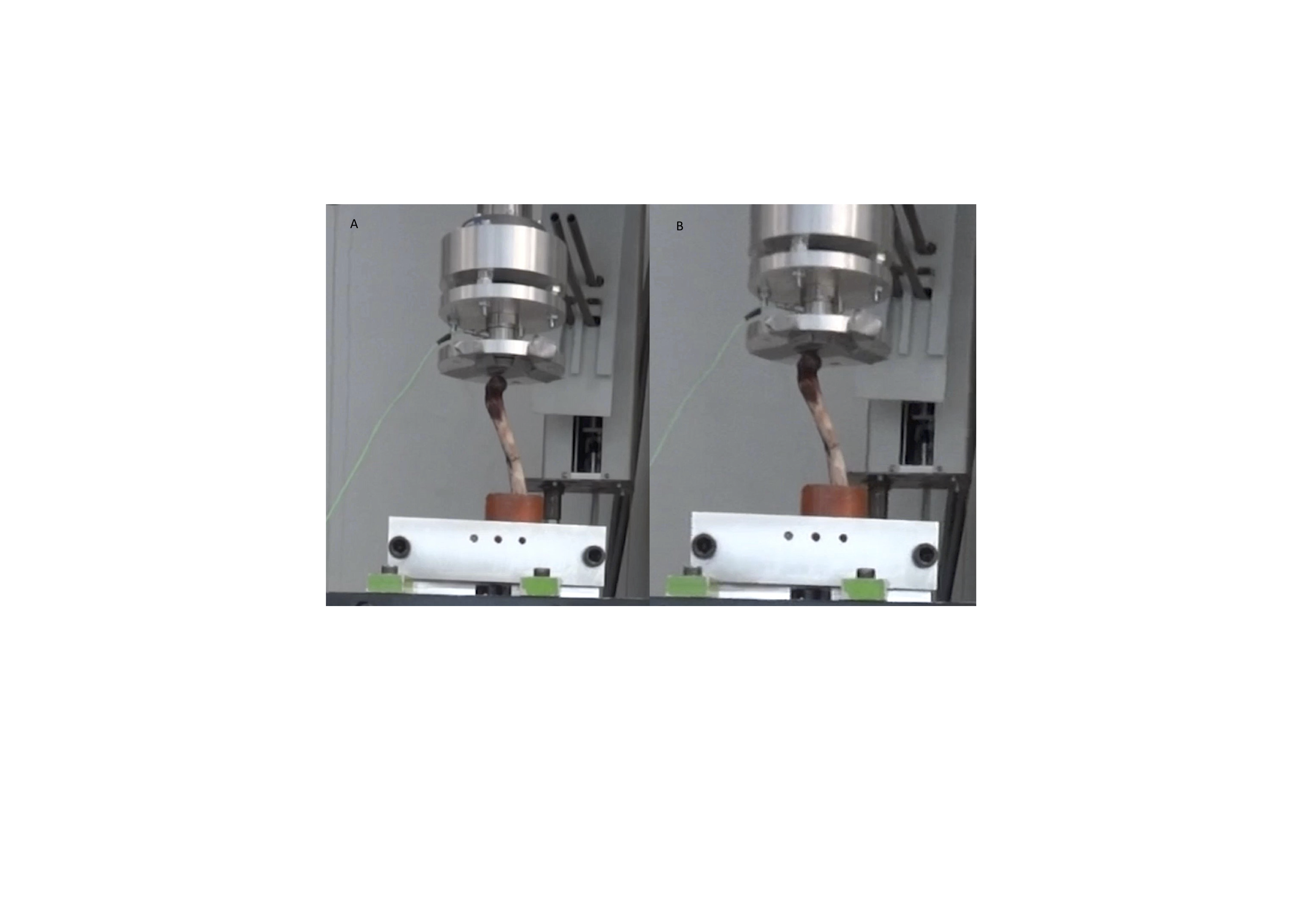

Supplement: S4 Fig — (A) This photograph shows the initial conformation of the femoral shape (specimen no. 18) as no compressive load was applied (starting point of the testing procedure). (B) This photograph shows a minor elastic deformation of the femur. It was taken when the physiological force has been successfully reached (time of the highest compressive load acting on the femur). Afterwards, the bone-nail construct shifted back into its original conformation with decreasing pressure (not shown in the photograph). (TIFF) [file pone.0231823.s006.tiff]

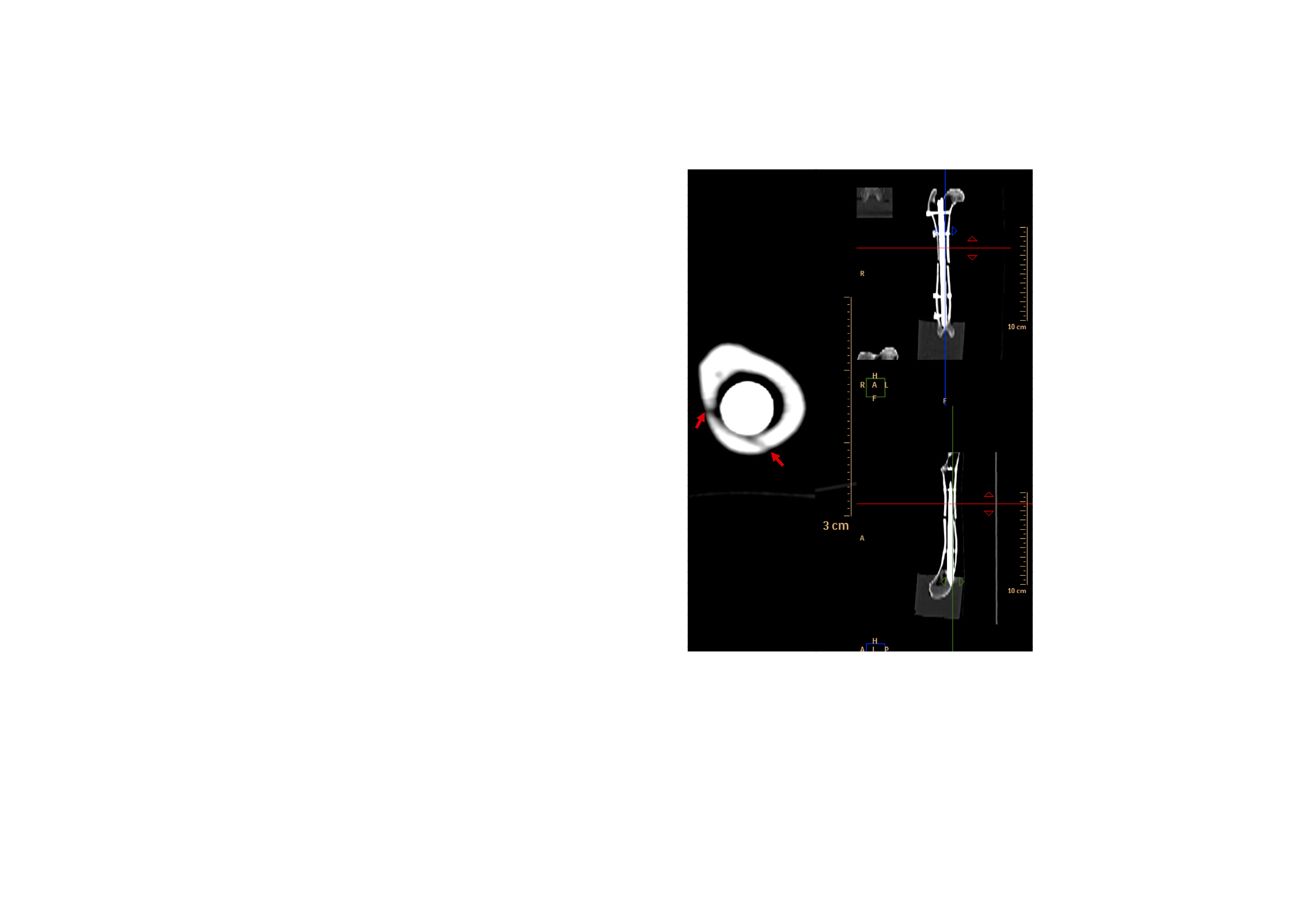

Supplement: S5 Fig — This CT-image illustrates two longitudinal fractures (red arrows) of a femur (specimen no. 30) that occurred due to bending testing. (TIFF) [file pone.0231823.s007.tiff]

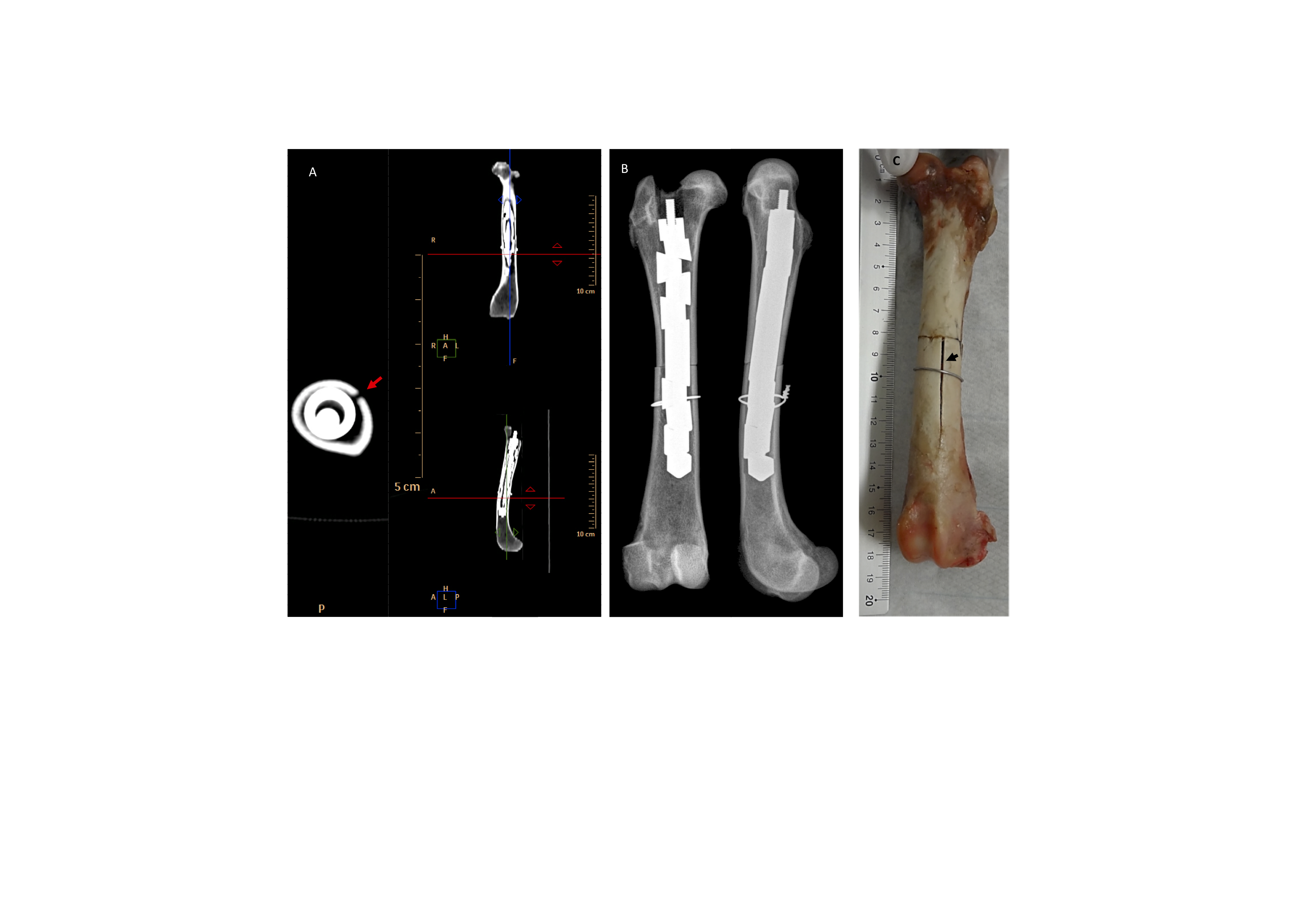

Supplement: S6 Fig — CT-image (A), biplanar radiographs (B) and a photograph (C) of an EXPN-bone construct after nail insertion. This figure illustrates the superiority of CT-images in detecting small longitudinal fractures compared to conventional radiographs. Therefore, in the CT-image (A) and photograph (C) of the femur, a longitudinal secondary femoral fracture is visible, whereas in both radiographs (B), no evidence of this fracture is given. (A) This CT-image represents the femur (specimen no. 8) with a secondary longitudinal fracture (red arrow) due to the expansion process of the nail, treated with a cerclage wire. (B) Caudocranial and mediolateral radiographs of the same secondary fractured femur, but without any radiographic evidence of the mentioned fracture. (C) A cranial photograph of the same femur, illustrating the mentioned secondary longitudinal femoral fracture (black arrow). (TIFF) [file pone.0231823.s008.tiff]
