# Supplementary material for: Biomechanical comparison of a new expandable intramedullary nail and conventional intramedullary nails for femoral osteosynthesis in dogs
Source: PLoS One. 2020 May 5;15(5):e0231823. doi: 10.1371/journal.pone.0231823 (PMC7200017; doi:10.1371/journal.pone.0231823)
Supplement: S1 Table — (DOCX) [file pone.0231823.s009.docx]

| Nail length  (mm) | 120 | 110 | 120 | 130 | 100 | 110 | 120 | 110 | 160 | 120 | 140 | 170 | 140 | 140 | 120 | 120 | 110 | 100 | 160 | 110 | 110 | 90 | 120 | 160 | 140 | 165 | 165 | 140 | 150 | 140 | 150 | 165 |
| --- | --- | --- | --- | --- | --- | --- | --- | --- | --- | --- | --- | --- | --- | --- | --- | --- | --- | --- | --- | --- | --- | --- | --- | --- | --- | --- | --- | --- | --- | --- | --- | --- |
| Nail diameter (mm) | 8 | 9 | 11 | 12 | 6 | 8 | 10 | 10 | 14 | 11 | 6 | 8 | 5 | 5 | 8 | 11 | 8 | 6 | 14 | 6 | 10 | 6 | 11 | 14 | 6 | 6 | 8 | 5 | 5 | 6 | 5 | 8 |
| Implant type^d^ | EXPN | EXPN | EXPN | EXPN | EXPN | EXPN | EXPN | EXPN | EXPN | EXPN | ILN | ILN | STMN | STMN | EXPN | EXPN | EXPN | EXPN | EXPN | EXPN | EXPN | EXPN | EXPN | EXPN | ILN | ILN | ILN | STMN | STMN | ILN | STMN | ILN |
| Fracture type | oblique | oblique | oblique | oblique | oblique | transverse | transverse | transverse | transverse | transverse | oblique | transverse | oblique | transverse | oblique | oblique | oblique | oblique | oblique | transverse | transverse | transverse | transverse | transverse | oblique | transverse | transverse | oblique | transverse | oblique | transverse | transverse |
| Femoral bowing^c^  (1-6) | 5 | 2 | 2 | 2 | 4 | 3 | 2 | 2 | 2 | 3 | 2 | 1 | 2 | 2 | 2 | 2 | 3 | 3 | 2 | 1 | 3 | 3 | 2 | 2 | 1 | 2 | 2 | 2 | 2 | 3 | 3 | 3 |
| Canal flare index (CFI) | 2.03 | 2.57 | 1.99 | 1.69 | 2.71 | 2.61 | 2.08 | 2.24 | 2.5 | 2.14 | 2.5 | 2.36 | 2.57 | 2.92 | 2.7 | 2.3 | 2.76 | 1.93 | 2.32 | 3.2 | 2.3 | 2.08 | 2.47 | 2.12 | 2.56 | 2.63 | 2.46 | 2.24 | 2.6 | 2.45 | 2.89 | 2.20 |
| Corticomedullary index (CMI) | 0.49 | 0.48 | 0.26 | 0.3 | 0.69 | 0.68 | 0.41 | 0.42 | 0.36 | 0.22 | 0.4 | 0.36 | 0.46 | 0.52 | 0.61 | 0.39 | 0.47 | 0.42 | 0.36 | 0.75 | 0.34 | 0.53 | 0.4 | 0.36 | 0.45 | 0.64 | 0.34 | 0.55 | 0.49 | 0.6 | 0.49 | 0.34 |
| Femoral isthmus diameter (mm) | 10 | 9.87 | 12.73 | 12.76 | 8.07 | 8.35 | 12.06 | 11.4 | 15.26 | 13.3 | 9.89 | 12.39 | 8.99 | 8.27 | 10.13 | 12 | 10.03 | 7.53 | 15.68 | 6.88 | 11.58 | 7.03 | 12.27 | 16.34 | 9.61 | 8.13 | 13.17 | 7.75 | 9.54 | 8.21 | 9.7 | 12.66 |
| Femur lenght (mm) | 227 | 179 | 221 | 223 | 188 | 206 | 210 | 178 | 270 | 216 | 189 | 206 | 172 | 172 | 191 | 214 | 207 | 146 | 280 | 173 | 204 | 161 | 197 | 260 | 178 | 204 | 227 | 174 | 194 | 172 | 198 | 206 |
| Osteophytes femoral neck^b^ (0-3) | 0 | 0 | 0 | 0 | 0 | 0 | 0 | 0 | 0 | 3 | 0 | 0 | 0 | 0 | 1 | 1 | 0 | 0 | 0 | 0 | 0 | 0 | 0 | 0 | 1 | 0 | 0 | 0 | 0 | 0 | 1 | 0 |
| Body weight (kg) | 39 | 30 | 30 | 30 | 31 | 34 | 35 | 29 | 50 | 37 | 30 | 32.2 | 29 | 22 | 39 | 36 | 30 | 8 | 68 | 17 | 24 | 13 | 32 | 50 | 35 | 24 | 35 | 22 | 22 | 24.2 | 36 | 32.2 |
| Age  (years) | 2.5 | 15 | 9 | 9 | 10 | 9 | ? | 8 | 7 | 13 | ? | 10 | ? | 11 | ? | 8 | 15 | 0.7 | 6 | ? | ? | 10 | ? | 7 | 10 | ? | 7 | ? | ? | 8 | 6 | 10 |
| Sex^a^ | ms | m | fs | fs | ms | ms | ms | ms | ms | f | f | ms | f | fs | ? | f | m | f | m | ? | f | fs | ms | ms | m | f | fs | f | ms | f | ms | ms |
| Breed | Mongrel dog | Mongrel dog | Rhodesian Ridgeback | Rhodesian Ridgeback | Australian Shepherd | Boxer | Mongrel dog | Chow Chow | Great Dane | Mongrel dog | Golden Retriever | Mongrel dog | Golden Retriever | Mongrel dog | Mongrel dog | Briard | Mongrel dog | Mongrel dog | Great Dane | Border Collie | German Shepherd | Mongrel dog | Dogo Argentionp | Great Dane | Elo | Irish Setter | Rhodesian Ridgeback | Mongrel dog | unknown | Labrador Retriever | Golden Retriever | Mongrel dog |
| Type of testing | Torsion | Torsion | Torsion | Torsion | Torsion | Torsion | Torsion | Torsion | Torsion | Torsion | Torsion | Torsion | Torsion | Torsion | Compression | Compression | Compression | Compression | Compression | Compression | Compression | Compression | Compression | Compression | Compression | Compression | Compression | Compression | Compression | Bending | Bending | Bending |
| No. | 1 | 2 | 3 | 4 | 5 | 6 | 7 | 8 | 9 | 10 | 11 | 12 | 13 | 14 | 15 | 16 | 17 | 18 | 19 | 20 | 21 | 22 | 23 | 24 | 25 | 26 | 27 | 28 | 29 | 30 | 31 | 32 |

^a^: Sex: m = male; ms = male spayed; f = female; fs = female spayed; ? = unknown

^b^: Degree of osteophytes presented at the femoral neck; 0 = none; 1 = minimal arthrosis; 2 = moderate arthrosis; 3 = severe arthrosis.

^c^: Degree of femoral curvature; from 1 = minimal femoral bowing to 6 = extremely bended femur.

^d^: EXPN: = expandable Nail; ILN = interlocking nail; STMN = Steinmann nail
