# Supplementary material for: Biomechanical comparison of a new expandable intramedullary nail and conventional intramedullary nails for femoral osteosynthesis in dogs
Source: PLoS One. 2020 May 5;15(5):e0231823. doi: 10.1371/journal.pone.0231823 (PMC7200017; doi:10.1371/journal.pone.0231823)
Supplement: S2 Table — (DOCX) [file pone.0231823.s010.docx]

| Fracture due to testing | no | no | yes | no | no | no | no | no | no | yes | no | yes | no | no | no | no | no | no | yes | No | yes | no | yes | yes | no | no | no | no | no | yes | no | yes |
| --- | --- | --- | --- | --- | --- | --- | --- | --- | --- | --- | --- | --- | --- | --- | --- | --- | --- | --- | --- | --- | --- | --- | --- | --- | --- | --- | --- | --- | --- | --- | --- | --- |
| Gap compression^m^ (0-6) | 0 | 0 | 0 | 0 | 0 | 0 | 0 | 0 | 0 | 0 | 0 | 0 | 1 | 5 | 0 | 0 | 0 | 0 | 0 | 4 | 0 | 0 | 0 | 0 | 0 | 0 | 0 | 6 | 6 | 0 | 0 | 0 |
| Fracture opening^l^  (0-6) | 1 | 0 | 1 | 1 | 3 | 0 | 0 | 0 | 0 | 0 | 0 | 0 | 0 | 0 | 0 | 0 | 2 | 0 | 1 | 0 | 0 | 0 | 0 | 0 | 0 | 0 | 0 | 0 | 0 | 0 | 1 | 0 |
| Shift of fracture ends^k^ (0-6) | 0 | 0 | 0 | 0 | 3 | 0 | 0 | 0 | 1 | 1 | 0 | 0 | 0 | 2 | 0 | 0 | 1 | 0 | 1 | 0 | 0 | 0 | 0 | 0 | 0 | 0 | 0 | 0 | 1 | 0 | 1 | 0 |
| Rotation of fracture ends^j^ (°) | 0 | 0 | 0 | 0 | 3 | 0 | 0 | 0 | 0 | 0 | 0 | 0 | 5 | 5 | 0 | 0 | 0 | 0 | 0 | 0 | 0 | 0 | 0 | 0 | 0 | 0 | 0 | 0 | 30 | 0 | 0 | 0 |
| Displacement^i^ (mm) | 4 | 4.3 | 2.2 | 3.9 | 7 | 2.6 | 4 | 1.7 | 3.7 | 5 | 3 | 1.6 | 10 | 4.5 | 7.5 | 3.2 | 8.6 | 1.9 | 7.8 | 9.4 | 6.9 | 7.8 | 3.5 | 5.9 | 3.7 | 5 | 5.8 | 13 | 8.5 | 4.2 | 6.2 | 5 |
| Deviation of the force^h^  (%) | 0 | 0 | 44 | 0 | 0 | 0 | 0 | 0 | 0 | 18 | 0 | 0 | 14 | 73 | 58 | 0 | 50 | 0 | 70 | 21 | 2 | 64 | 69 | 69 | 0 | 0 | 0 | 95 | 19 | - | - | - |
| Resisted force^g^ | 1.9 Nm | 1.5 Nm | 0.84 Nm | 1.5 Nm | 1.5 Nm | 1.7 Nm | 1.7 Nm | 1.4 Nm | 2.5 Nm | 1.48 Nm | 1.5 Nm | 1.6 Nm | 1.2 Nm | 0.3 Nm | 320 N | 689 N | 290 N | 149 N | 400 N | 260 N | 455 N | 90 N | 190 N | 300 N | 677 N | 464 N | 673 N | 20 N | 340 N | - | - | - |
| Physiological force^f^ | 1.9 Nm | 1.5 Nm | 1.5 Nm | 1.5 Nm | 1.5 Nm | 1.7 Nm | 1.7 Nm | 1.4 Nm | 2.5 Nm | 1.8 Nm | 1.5 Nm | 1.6 Nm | 1.4 Nm | 1.1 Nm | 755 N | 689 N | 580 N | 149 N | 1316 N | 329 N | 464 N | 253 N | 619 N | 967 N | 677 N | 464 N | 673 N | 426 N | 422 N | - | - | - |
| Gap existent^e^ | no | no | no | no | no | no | no | no | no | no | no | yes | no | no | no | no | no | no | no | yes | no | yes | yes | yes | yes | yes | no | yes | yes | no | no | yes |
| Fracture  reposition^d^  (1-6) | 2 | 1 | 2 | 2 | 2 | 2 | 1 | 1 | 1 | 2 | 2 | 4 | 3 | 2 | 1 | 1 | 1 | 2 | 1 | Gap maintained | Gap maintained | Gap maintained | Gap maintained | Gap maintained | Gap maintained | Gap maintained | Gap maintained | Gap maintained | Gap maintained | 2 | 3 | 4 |
| Shortening of the nail^c^  (mm) | 5.9 | 1.51 | 0.85 | 1.8 | 6.63 | 1.66 | 5.01 | 1.74 | 6.11 | 5.82 | - | - | - | - | 7.13 | 5.12 | 6 | 5.05 | 10.02 | 6.39 | 4.51 | 3.83 | 1.86 | 6.17 | - | - | - | - | - | - | - | - |
| Nail diameter increase^b^ (%) | 37 | 27 | 27 | 23 | 38 | 36 | 33 | 41 | 32 | 40 | - | - | - | - | 39 | 30 | 30 | 38 | 35 | 37 | 40 | 29 | 42 | 39 | - | - | - | - | - | - | - | - |
| Implant type^a^ | EXPN | EXPN | EXPN | EXPN | EXPN | EXPN | EXPN | EXPN | EXPN | EXPN | ILN | ILN | STMN | STMN | EXPN | EXPN | EXPN | EXPN | EXPN | EXPN | EXPN | EXPN | EXPN | EXPN | ILN | ILN | ILN | STMN | STMN | ILN | STMN | ILN |
| Type of testing | Torsion | Torsion | Torsion | Torsion | Torsion | Torsion | Torsion | Torsion | Torsion | Torsion | Torsion | Torsion | Torsion | Torsion | Compression | Compression | Compression | Compression | Compression | Compression | Compression | Compression | Compression | Compression | Compression | Compression | Compression | Compression | Compression | Bending | Bending | Bending |
| No. | 1 | 2 | 3 | 4 | 5 | 6 | 7 | 8 | 9 | 10 | 11 | 12 | 13 | 14 | 15 | 16 | 17 | 18 | 19 | 20 | 21 | 22 | 23 | 24 | 25 | 26 | 27 | 28 | 29 | 30 | 31 | 32 |

^a^: EXPN = expandable Nail; ILN = interlocking nail; STMN = Steinmann nail

^b^: Percentage of nail diameter increase compared to the initial (non-expanded) size; e.g. 50 % increase in an initially ø 10 mm nail would lead to a nail size of ø 15 mm (after nail expansion).

^c^: Shortening (in mm) of the nail due to the expansion process.

^d^: Quality of fracture reduction; 1 = nearly anatomical reduction of the fracture ends to 6 = high degree of fragment displacement.

^e^: Presence of a continuous femoral gap after nail expansion.

^f^: The calculated physiological forces (compressive (N) or torsional (Nm) load) acting on the femoral bone in normal motion for each individual specimen.

^g^: The maximum load (compressive or torsional forces) that the specimens successfully resisted during biomechanical testing.

^h^: Displays the percentage deviation between the calculated physiological force and the actual resisted force.

^i^: Displacement/shortening of the testing device including the femoral bone.

^j^: Degree of rotation of the bone segments at the fracture site present after testing.

^k^: Shift of the bone segments at the fracture site present after testing; 0 = none; 1 = minimal to 6 = high degree of shifting .

^l^: Opening of the osteotomy gap present after testing; 0 = none; 1 = minimal to 6 = high degree of opening.

^m^: Gap compression present after testing; 0 = none; 1 = minimal to 6 = high degree of gap compression.
